# Bioinformatics Analysis of Small RNAs in Pima (*Gossypium barbadense* L.)

**DOI:** 10.1371/journal.pone.0116826

**Published:** 2015-02-13

**Authors:** Hongtao Hu, Dazhao Yu, Hong Liu

**Affiliations:** 1 Center for Bio-Pesticide Research, Hubei Academy of Agricultural Sciences, Wuhan, Hubei, China; 2 Department of Biological Engineering, Hubei Vocational College of Biological Sciences and Technology, Wuhan, Hubei, China; 3 Institute of Plant Protection & Soil Fertilizer, Hubei Academy of Agricultural Sciences, Wuhan, Hubei, China; 4 College of Life Sciences, Hunan University of Arts and Sciences, Changde, Hunan, China; 5 College of Fisheries, Huazhong Agricultural University, Wuhan, Hubei, China; Nanjing Agricultural University, CHINA

## Abstract

Small RNAs (sRNAs) are ~20 to 24 nucleotide single-stranded RNAs that play crucial roles in regulation of gene expression. In plants, sRNAs are classified into microRNAs (miRNAs), repeat-associated siRNAs (ra-siRNAs), phased siRNAs (pha-siRNAs), *cis* and *trans* natural antisense transcript siRNAs (*cis*- and *trans*-nat siRNAs). Pima (*Gossypium barbadense* L.) is one of the most economically important fiber crops, producing the best and longest spinnable fiber. Although some miRNAs are profiled in Pima, little is known about siRNAs, the largest subclass of plant sRNAs. In order to profile these gene regulators in Pima, a comprehensive analysis of sRNAs was conducted by mining publicly available sRNA data, leading to identification of 678 miRNAs, 3,559,126 ra-siRNAs, 627 pha-siRNAs, 136,600 *cis*-nat siRNAs and 79,994 *trans*-nat siRNAs. The 678 miRNAs, belonging to 98 conserved and 402 lineage-specific families, were produced from 2,138 precursors, of which 297 arose from introns, exons, or intron/UTR-exon junctions of protein-coding genes. Ra-siRNAs were produced from various repeat loci, while most (97%) were yielded from retrotransposons, especially LTRs (long terminal repeats). The genes encoding auxin-signaling-related proteins, NBS-LRRs and transcription factors were major sources of pha-siRNAs, while two conserved *TAS*3 homologs were found as well. Most *cis*-NATs in Pima overlapped in enclosed and convergent orientations, while a few hybridized in divergent and coincided orientations. Most *cis*- and *trans*-nat siRNAs were produced from overlapping regions. Additionally, characteristics of length and the 5’-first nucleotide of each sRNA class were analyzed as well. Results in this study created a valuable molecular resource that would facilitate studies on mechanism of controlling gene expression.

## Introduction

Small RNAs (sRNAs) are short, single-stranded RNAs that silence gene expression transcriptionally or post-transcriptionally [1–3], which is also known as RNA-induced silencing. sRNAs are produced from longer, double stranded RNA (dsRNA) precursors by DICER-LIKE proteins (DCLs) in plants [4]. Mature sRNAs are loaded into Argonaute (AGO) proteins to form RNA inducing complex in which sRNAs serve as guides to complementarily bind target RNA molecules, leading to degradation or translation repression of the targets [5,6]. In addition, some sRNAs are able to induce methylation or histone modification of target genomic loci as well [7–9].

Generally, sRNAs are classified into microRNAs (miRNAs) and small interfering RNAs (siRNAs), according to their origin and biogenesis [10]. miRNAs are processed from hairpin-shaped RNA precursors (pre-miRNAs) that are transcribed by RNA polymerase II [11]. In plants, most miRNAs are derived from non-coding genes, whose transcripts are further processed by DCLs with presence of the zinc finger protein SERRATE [12], HYPONASTIC LEAVES1 [13,14], the G-patch domain protein TOUGH [15] and hnRNP-like protein [16]. However, recent studies show that miRNAs can also be produced from introns or exon-intron junctions of protein-coding genes [17,18], which are regulated through alternative splicing (AS) [19]. In plants, canonical miRNAs are 21 nt in length and regulate gene expression transcriptionally [20,21]. Many miRNAs target transcription factors, such as SQUAMOSA Promoter Binding Protein-Like, MYB and HD-ZIP [22,23], which are crucial for plant growth, development and stress response [21,24,25], but the so-called long miRNAs (24-nt in length) function to direct DNA methylation [8]. In plants, siRNAs are produced from double stranded RNAs (dsRNAs), whose synthesis requires activities of RNA polymerase IV/V (Pol IV/V) [26,27] and RNA-dependent RNA polymerases (RDRs) [28,29]. siRNAs can be sub-divided into four groups, repeat-associated siRNAs (ra-siRNAs), phased siRNAs (pha-siRNAs), *cis*- and *trans* natural antisense siRNAs (*cis*- and *trans*-nat siRNAs) [10]. Ra-siRNAs, typically 24 nt in length, are also known as heterochromatic siRNAs (hc-siRNAs) transcribed from repeat DNA loci by RNA polymerase IV (Pol IV) [30] with assistance of certain proteins, such as CLASSY1 [31], SHH1[32] and DTF1[33]. Ra-siRNAs are the biggest subclass of plant sRNAs [34] and mediate epigenetic modification through DNA methylation [7]. Pha-siRNAs are plant-specific sRNAs cleaved from pha-siRNA-yielding transcripts (PYTs) in phased manner, by which sRNAs are produced precisely in a head-to-tail arrangement [35,36]. A subset of pha-siRNAs that can function in *trans* to repress gene expression at transcriptional level are termed as *trans*-acting siRNAs (ta-siRNAs), and the transcripts producing ta-siRNAs are called *TAS*s [10,35,37]. To date, *TAS*3 homologs are widely found in various plants, and their ta-siRNAs are shown to play crucial roles in regulation of plant developmental timing and stress response [36–38]. Both *cis*- and *trans*-nat siRNAs are processed from natural antisense transcripts (NATs) by DCL proteins in plants [10,39] but differ by their origin. Generally, *cis*-NATs are transcribed from the same genomic loci with opposite polarities, whereas *trans*-NATs are generated from different genomic loci. Nat-derived siRNAs are shown to regulate diverse physiological processes, such as fertilization and stress response to environmental cues [7,39–42].

Pima (*Gossypium barbadense* L.) is one of the most important fiber crops worldwide, belonging to allotetraploid cotton that is evolved through hybridization and subsequent polyploidization events between the A-genome and D-genome cottons, closely related to *Gossypium arboreum* L. (A2) and *Gossypium raimondii* L. (D5), respectively [43,44]. The merger of the A and D genome species into allotetraploid species led to superior fiber length and quality, and sRNAs are shown to play crucial roles in regulation of fiber development [45–50]. Pang et al. discover a total of 31 miRNA families as well as their targets expressed in vegetative and reproductive tissues [51]. Analysis of deep sequencing data shows that a large number of known and novel miRNA families are expressed in the Upland cotton ovule and fiber, and a subset of them displays differential expression patterns [49–52]. For instance, miR166 and miR172 are expressed abundantly in the fiber, but miR828, miR475 and miR1023 are expressed at extremely low levels [50]. Comparative analysis of sRNAs reveals significant differences in miRNAs expression between the wild type Upland cotton and its fuzzless-lintless mutant [53]. Additionally, miR828 and miR858 targeting *GhMYB2D* mRNA leads to generation of ta-siRNAs, mediating fiber development [47]. In addition to miRNAs, *TAS*3-derived ta-siRNAs triggered by miR390 are found in Upland cotton as well [54]. The suppression of miR156/157 leads to the reduction of mature fiber length in Pima [48]. Compared to Upland cotton, apparently fewer miRNAs are identified in Pima [48,55], which produces the best and longest textile fiber, and siRNAs being the most abundant sRNA class in plants [34] still remain largely unknown. To profile these gene regulators in Pima, we analyzed two publicly available sRNA datasets from the root and fiber, resulting in identification of 5 major sRNA species. This is the first comprehensive analysis of Pima sRNAs and created a valuable molecular resource, which largely expands the scope of sRNAs in cotton and is very crucial for future studies on mechanism of regulating plant growth and development.

## Methods and Materials

### Small RNA datasets of *G*. *barbadense*

Two sRNA datasets of Pima prepared from the root (GSM699076) and 10 DPA (days post anthesis) fiber (GSM634227) were downloaded from NCBI Gene Expression Omnibus (http://www.ncbi.nlm.nih.gov/geo). The two datasets contain 15,599,325 and 7,764,225 reads (S1 Table), respectively. For further analysis, sequences 18 to 26 nt in length were retained, leaving a total of 9,518,811 unique sRNAs, and the total reads of each sRNA from the two datasets were recorded for expression-based filtration, using in-house Perl scripts.

### The cotton nucleotide sequence dataset

Due to unavailability of the allopolyploid cotton genome, a comprehensive cotton nucleotide dataset was collected and assembled for this study, including cotton A2 [44] and D5 genomes [43], and cotton-derived transcripts, downloaded from NCBI (ftp://ftp.ncbi.nlm.nih.gov/repository/UniGene/), PlantDB (http://www.plantgdb.org), CottonGen (http://www.cottongen.org), Comparative Evolution of Genomics of Cotton (http://128.192.141.98/CottonFiber), and Cotton Gene Index (version 11, http://compbio.dfci.harvard.edu).

### Identification of conserved and lineage-specific miRNAs

To identify miRNAs, sRNAs with a total abundance ≥10 reads were submitted to a modified miREAP (http://sourceforge.net/projects/mireap/), in which 3 nucleotide shifts from putative miRNA loci are allowed, using the following parameters: the maximal distance between miRNAs and miRNA*s (-d 450), the maximal copies on the genome (-u 5000), and defaults for others. The putative miRNAs obtained from miREAP were further filtered by strand bias (≥0.8), which was calculated by dividing reads from sense strand by total reads, and abundant bias (≥0.6), which was calculated by dividing total reads of top 3 sRNAs from miRNA loci by total reads [56,57]. Additionally, the remaining putative miRNAs were filtered by the secondary structures of their pre-miRNAs in which maximal four mismatches are allowed between miRNAs and miRNA*s [57], using CentroidFold [58]. The identified miRNAs that are homologous to known plant miRNAs in miRBase version 21 (http://www.mirbase.org) with three or less substitutions are classified into conserved miRNAs (cs-miRNAs), while the others are considered as candidates of lineage-specific miRNAs (ls-miRNAs) [10].

To determine miRNAs yielded from protein-coding genes, cotton-derived transcripts were mapped to the cotton A2 and D5 genomes, using Blastn [59,60] with e-value < 10^-6^. Those transcripts, which were mapped to upstream and/or downstream of miRNA loci within 5000 nt with ≥96% identity, ≤3 mismatches and no indel (insertion-deletion), were selected for further analysis. The transcripts mapped to miRNA loci were annotated using Blastx [59,60] against plant-derived proteins deposited in NCBI (http://www.ncbi.nlm.nih.gov/) with a stringent e-value <10^-6^. miRNAs derived from protein-coding genes were further classified into 3 classes based on the location of their pre-miRNAs, intron-derived miRNAs processed from introns of protein-coding genes [17], exon-derived miRNAs cleaved from exons of protein coding genes, and junction-derived miRNAs yielded from exon-intron/UTR junctions [18].

### Identification of ra-siRNAs

To determine ra-siRNAs, we extracted repeat DNAs from the cotton A2 and D5 genomes [43,44]. Given that the information of repeat loci in the cotton A2 genome is not publicly available, we identified repeat DNA loci in the A2 genome using RepeatMasker [61] with default parameters. All sRNAs matching repeat DNAs were considered as ra-siRNAs.

### Identification of pha-siRNAs

In order to identify pha-siRNAs, sRNAs (excluding miRNAs and ra-siRNAs) were subjected to pha-siRNA analysis, using UEA Small RNA WorkBench [62] with p-value <10^-3^ that employs the algorithm described previously [63]. The putative PYTs obtained from UEA Small RNA WorkBench [62] were further filtered by three extra characteristics: 1). the PYTs produced at least two pha-siRNAs; 2). at least a pha-siRNA had a total read count ≥5; 3). the ratio of pha-siRNAs/non-pha-siRNAs was ≥0.6. Analysis of miRNA initiators triggering the synthesis of pha-siRNAs was conducted, referring to the methodology described in early study [64] with minor modification. Briefly, putative initiators were predicted using psRNATarget with length for complementarity scoring (18) and expectation value (4.0). Then, the miRNAs that were predicted to flank the upstream or downstream of pha-siRNAs within 148 nt were selected for this analysis, allowing one nucleotide shift from the classic cleavage site [64].

### Identification of *cis-* and *trans*-nat siRNAs

The *cis*-nat siRNAs were determined, referring to the methods described previously [65]. Briefly, cotton-derived transcripts were mapped to both cotton A2 and D5 genomes, and then pairs of transcripts derived from the same genomic loci with at least 50 nt overlap but from different orientations were chosen as *cis*-NATs. sRNAs produced from *cis*-NATs were considered as *cis*-nat siRNA candidates.

After excluding *cis*-NATs, the remaining transcripts were used for analysis of *trans*-NATs. Putative *trans*-NATs were determined, using Blastn search for pairs of sequences that were complementary to each other with at least 100 nt overlap. The putative *trans*-NATs were then filtered by secondary structure using RNAcoFold in Vienna RNA package [66]. Those putative *trans*-NATs that can form at least 50 nt dsRNAs, in which 90% nucleotides were base-paired, were considered as *trans*-NATs, and sRNAs matched to *trans*-NATs were *trans*-nat siRNA candidates.

## Results and Discussion

### Conserved and lineage-specific miRNAs in Pima

An analysis of the Pima sRNA datasets (S1 Table), which contained 9,518,811sRNAs, was performed. This led to identification of a total of 678 miRNAs from 2,138 pre-miRNAs (S2 Table), of which 222 and 456 were assigned to 98 cs-miRNA and 402 ls-miRNA families, respectively.

A comparison of the 98 Pima cs-miRNA families across plants was conducted (Table 1). Twenty three (23%) cs-miRNA families were present in both Eudicotyledon and Monocotyledon, with a subset also found in Embryophyta and/or Coniferophyta, suggesting that they have common ancient origins [67]. Notably, miR477 was a special family that was present in both Embryophyta and Eudicotyledons, but absent in Monocotyledon. Ten (10%) families were detected in only Eudicotyledons, indicating that they are Eudicotyledon-specific, and the other 64 families (65%) were restricted to Malvaceae, reflecting that they are poorly conserved.

**Table 1:** Conserved miRNA families of Pima across plants.

miRNAs	Coniferophyta	Embryophyta	Magnoliophyta																	
			Eudicotyledons																	Monocotyledons
			Araliaceae	Asteraceae	Brassicaceae	Caricaceae	Cucurbitaceae	Euphorbiaceae	Fabaceae	Lamiales	Linaceae	Malvaceae	Ranunculaceae	Rhizophoraceae	Rosaceae	Rutaceae	Salicaceae	Solanaceae	Vitaceae	
miR156	+	+		+	+	+	+	+	+	+	+	+	+	+	+	+	+	+	+	+
miR396	+	+		+	+	+	+	+	+	+	+	+	+	+	+	+	+	+	+	+
miR160	+	+		+	+	+	+	+	+	+	+	+	+		+	+	+	+	+	+
miR166	+	+		+	+	+	+	+	+	+	+	+	+		+	+	+	+	+	+
miR171	+	+		+	+	+	+	+	+	+	+	+	+		+	+	+	+	+	+
miR395	+	+		+	+	+	+	+	+	+	+	+	+		+	+	+	+	+	+
miR159	+	+		+	+	+	+	+	+		+	+	+		+	+	+	+	+	+
miR167		+		+	+	+	+	+	+	+	+	+	+		+	+	+	+	+	+
miR169	+			+	+	+	+	+	+	+	+	+	+		+	+	+	+	+	+
miR164	+			+	+	+	+	+	+	+	+	+			+	+	+	+	+	+
miR172				+	+	+	+	+	+	+	+	+	+		+	+	+	+	+	+
miR390	+	+		+	+	+	+	+	+		+	+			+	+	+	+	+	+
miR394				+	+	+	+	+	+	+	+	+			+	+	+	+	+	+
miR162	+			+	+	+	+	+	+		+	+			+	+	+	+	+	+
miR168				+	+		+	+	+		+	+	+		+	+	+	+	+	+
miR393				+	+	+	+	+	+		+	+			+	+	+	+	+	+
miR397	+				+		+	+	+	+	+	+			+	+	+	+	+	+
miR399				+	+		+	+	+	+	+	+	+		+	+	+	+	+	+
miR482	+		+					+	+			+	+		+	+	+	+	+	+
miR530				+			+	+	+		+	+	+		+	+	+	+		+
miR535	+	+				+		+				+	+		+	+			+	+
miR827					+			+				+			+	+	+	+		+
miR1877												+								+
miR477		+				+	+	+				+	+		+	+	+	+	+	
miR403				+	+			+	+			+			+	+	+	+	+	
miR2111					+		+	+	+			+			+		+		+	
miR3627												+			+		+	+	+	
miR1446								+				+					+	+		
miR2950								+				+							+	
miR479												+				+		+	+	
miR170					+							+								
miR3954												+				+				
miR6140			+									+								
miR8148						+						+								
miR2947												+								
miR2948												+								
miR2949												+								
miR3267												+								
miR3476												+								
miR7484												+								
miR7486												+								
miR7489												+								
miR7491												+								
miR7492												+								
miR7494												+								
miR7495												+								
miR7498												+								
miR7499												+								
miR7500												+								
miR7501												+								
miR7502												+								
miR7504												+								
miR7505												+								
miR7506												+								
miR7507												+								
miR7508												+								
miR7510												+								
miR7512												+								
miR7513												+								
miR8632												+								
miR8633												+								
miR8634												+								
miR8635												+								
miR8636												+								
miR8637												+								
miR8638												+								
miR8639												+								
miR8641												+								
miR8642												+								
miR8644												+								
miR8646												+								
miR8675												+								
miR8677												+								
miR8680												+								
miR8695												+								
miR8701												+								
miR8702												+								
miR8706												+								
miR8709												+								
miR8721												+								
miR8722												+								
miR8727												+								
miR8729												+								
miR8732												+								
miR8733												+								
miR8740												+								
miR8746												+								
miR8752												+								
miR8753												+								
miR8758												+								
miR8762												+								
miR8764												+								
miR8766												+								
miR8767												+								
miR8768												+								
miR8775												+								
miR8783												+								
miR8785												+								

*: Each plus sign “+” represents the presence of miRNAs in corresponding plants.

**Figure 1:**
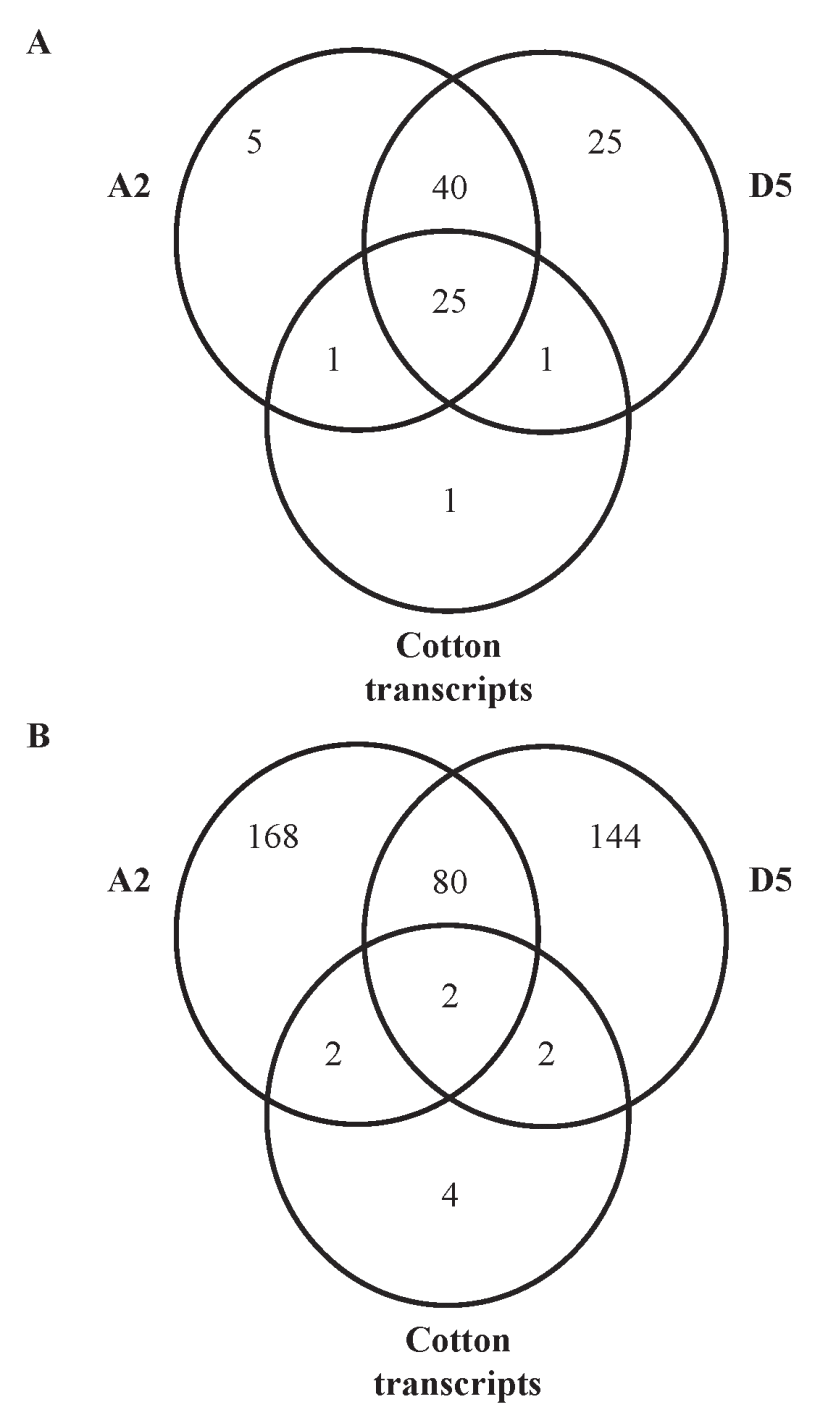
Venn diagram shows the number of miRNAs derived from different originations. The numbers of miRNAs of Pima derived from different originations are shown in A (conserved miRNAs) and B (lineage-specific miRNAs), respectively. A2, D5 and cotton transcripts represent the number of miRNA family detected from A2 genome (*G*. *arbroeum)*, D5 genome (*G*. *raimondii*) and cotton transcripts, respectively.

Among the 98 cs-miRNA families, 25 (26%) were detected from all three reference resources (Fig. 1A), 40 (41%) were shared between A2 and D5 genomes but not detected from cotton transcripts, while two were shared between either of two genomes and cotton transcripts. In addition, 5, 25 and 1 cs-miRNA families were uniquely detected from A2, D5 genome, and cotton transcripts, respectively. By contrast, only two ls-miRNA families (0.5%) were detected in all three reference resources (Fig. 1B), 80 (20%) were shared between A2 and D5 genome but not found in cotton transcripts, while 168 (42%), 144 (36%) and 4 (1%) were uniquely detected from A2, D5 genome, and cotton transcripts, respectively.

**Figure 2:**
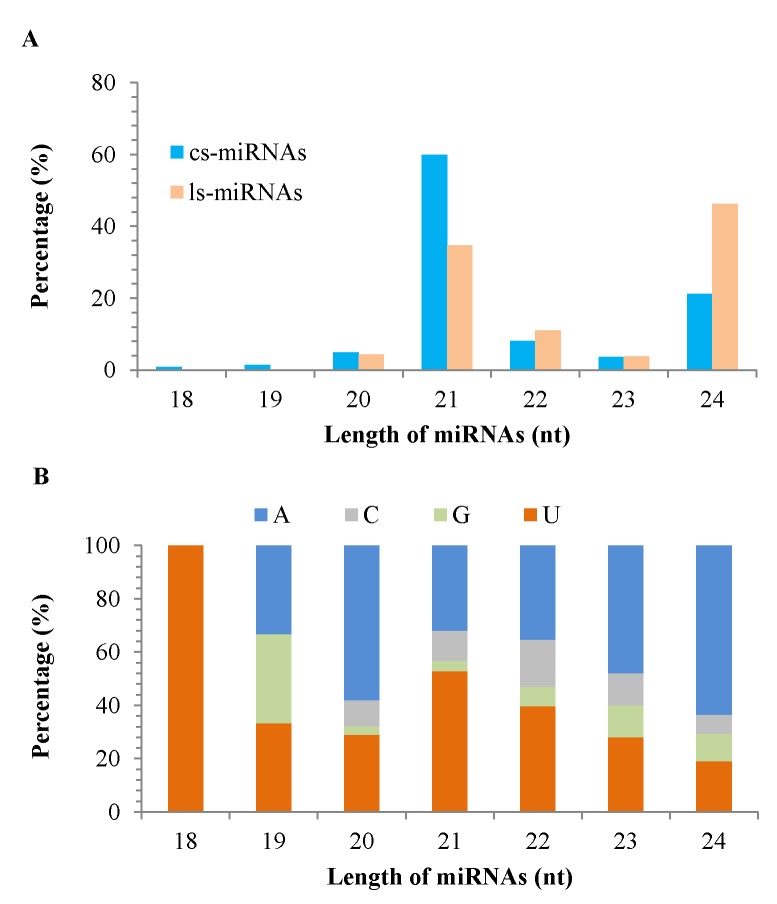
Characteristics of Pima miRNAs. The length distribution of Pima miRNAs is shown in A, and the 5’end nucleotide distribution of Pima miRNAs is shown in B.

Pima miRNAs varied from 18 to 24 nt in length, with almost all miRNAs (99%) being 20 to 24 nt in length (Fig. 2A). Most cs-miRNAs (60%) were 21 nt in length, whereas nearly half ls-miRNAs (46%) were 24 nt in length. In plants, miRNAs are predominantly 21 nt in length and regulate gene expression post-transcriptionally [10,68], but long miRNAs (24 nt in length) function to direct chromatin modification and consequently result in transcriptional repression of target loci [8]. Cs-miRNAs and ls-miRNAs displayed different length characteristics, which might be associated with their regulatory roles [8,69].

Analysis of the 5’first nucleotide revealed a higher frequency of A or U and a less frequency of C or G at the 5’end of Pima miRNAs (Fig. 2B). The 5’-first nucleotide is important for sorting sRNAs into different AGO clades, and changing 5’-first nucleotide of a miRNA can redirects it to a different AGO complex and alter its biological activity [5]. Thus, it could be inferred that the characteristic of the 5’-first nucleotide is also functionally important for miRNAs in Pima.

Compared to previous studies in Pima and other cotton species [48–50,55], more conserved and lineage-specific miRNAs along with their pre-miRNAs are identified in Pima in this study, which largely expands cotton miRNA information. Consistent with previous study [67], most miRNAs are not conserved among plants, which are proposed to play species-specific roles in plant growth and development [70].

### miRNAs derived from protein-coding genes

**Figure 3:**
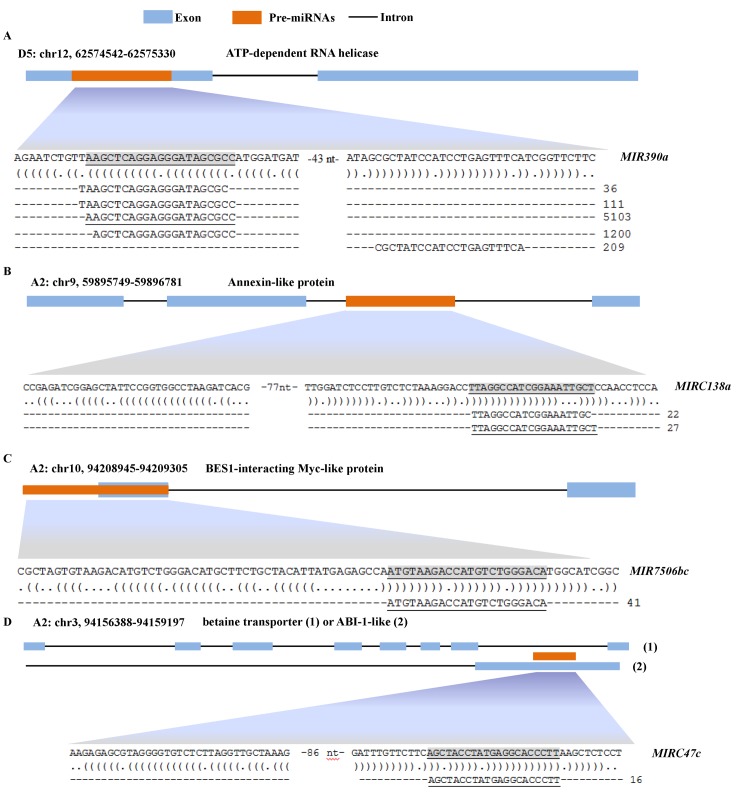
Examples of four classes of protein-gene-derived pre-miRNAs. Four classes of protein-gene-derived pre-miRNAs are shown in A (exon-derived), B (intron-derived), C (junction-derived), and D (intron/exon-derived). The nucleotide sequences represent the pre-miRNAs, in which the shaded and underlined nucleotides represent miRNA sequences. The secondary structure of pre-miRNAs is shown below each pre-miRNA. The sRNAs mapped to each pre-miRNAs are shown below the secondary structure and the reads of each sRNAs is shown on the right.

Typically, miRNAs in plants are produced from non-protein-coding genes [10,57], while recent studies show that miRNAs can also be produced from protein-coding genes in *Arabidopsis* and rice [71,72]. In the present study, 297 pre-miRNAs were identified to arise from introns, exons, or intron-exon junctions of protein-coding genes (S3 Table). These pre-miRNAs were able to form well-defined hairpin structures of plant miRNAs (Fig. 3A to 3D) [56,57]. Notably, a special pre-miRNA (*MIR*C47c) was observed, which could be processed from either an intron or exon of two different protein-coding genes from the same genomic loci (Fig. 3D), indicating a potential AS site.

In animals, the biogenesis of miRNAs derived from exon-intron junctions is negatively regulated through AS, which is competitive with Drosha cleavage [19]. In *Arabidopsis*, an AS event that occurs in the intron where *MIR*400 is located is specifically induced by heat stress, providing the evidence that AS acts as a regulatory mechanism linking miRNAs and environmental stress in plants [72].

### Identification of ra-siRNAs in Pima

To identify ra-siRNAs, sRNAs (excluding miRNAs) were mapped to repeat DNA loci in both cotton A2 [43] and D5 [44] genomes. Consequently, 3,559,126 sRNAs were mapped to repeat DNA loci, which were considered as ra-siRNA candidates (S4 Table). These ra-siRNAs were further classified into 5 different types, according to their origin (Table 2). A total of 3,447,580 ra-siRNAs arose from retrotransposons, of which 79% were derived from LTRs (long terminal repeats). Except for rRNA-derived ra-siRNAs, approximately 75% ra-siRNAs were uniquely detected in either A2 or D5 genome, indicating that ra-siRNAs are not well conserved between the two genomes.

**Table 2:** Component of ra-siRNAs of *G*. *barbadense*.

Type	Cotton Sub-genome		
	A2	D5	Both A2 and D5	Total
Transposon	31,102	49,934	14,973	66,063
DNA/MuDR	21,050	30,748	9,794	42,004
DNA/En-Spm	6,144	12,550	3,305	15,389
DNA	2,636	4,830	1,304	6,162
DNA/hAT	1,150	1,549	486	2,213
DNA/Harbinger	42	152	32	162
DNA/TcMar	80	96	52	124
DNA/Mite	0	6	0	6
RC/Helitron	0	3	0	3
Retrotransposon	2,091,427	2,213,611	857,458	3,447,580
LTR	796,849	922,373	343,074	1,376,148
LTR/Gypsy	737,565	536,980	282,674	991,871
LTR/Copia	216,186	227,997	89,959	354,224
Non LTR	37,724	56,264	18,520	75,468
Retroelement	301,759	467,958	122,859	646,858
LINE	1,344	2,037	372	3,009
SINE	0	2	0	2
rRNA	25,763	22,994	20,772	27,985
Simple repeat	15,852	7,133	5,641	17,344
Others	14	148	8	154
Total	2,164,158	2,293,820	898,852	3,559,126

**Figure 4:**
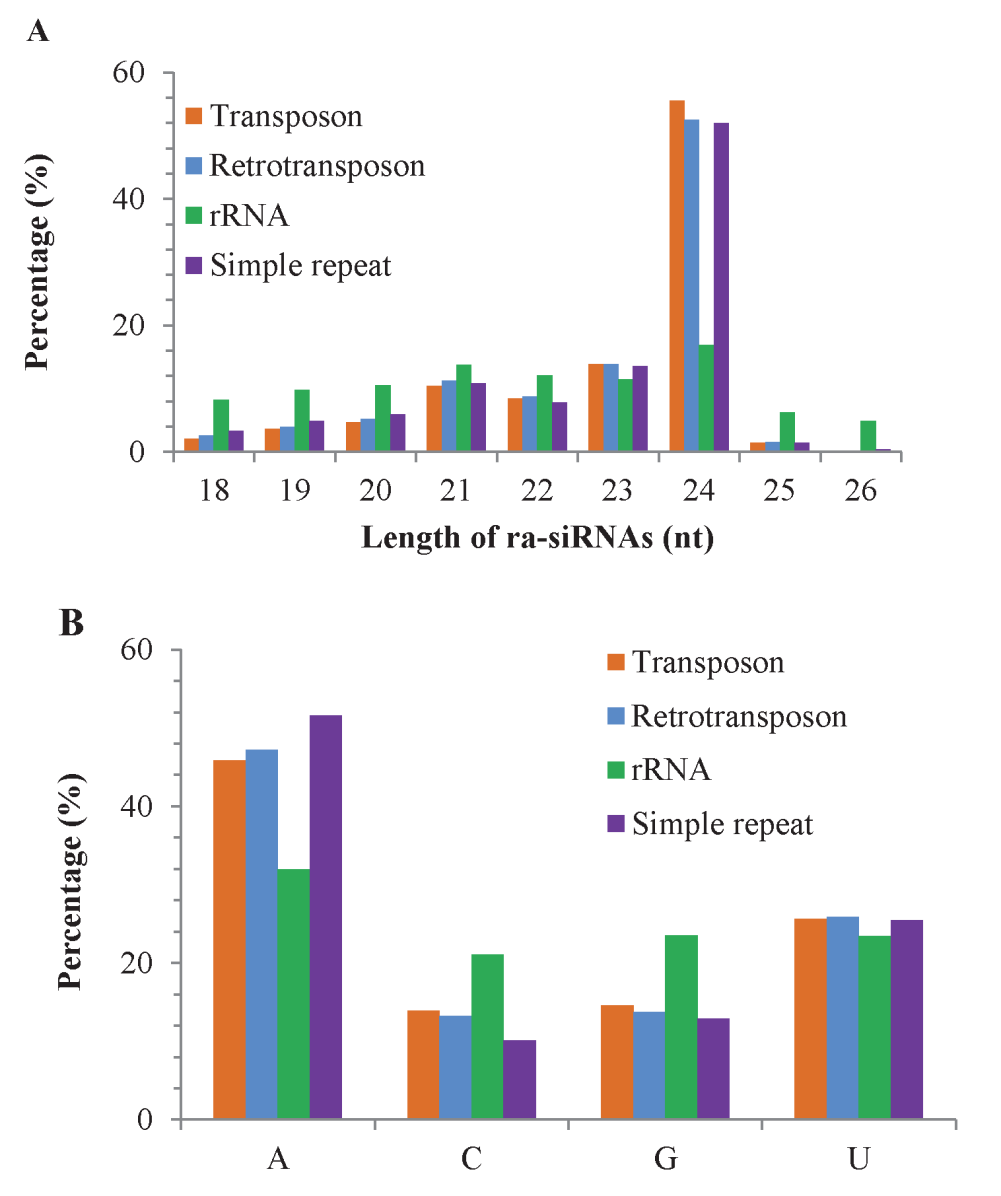
Characteristics of ra-siRNAs in *G*. *barbadense*. The length distribution of ra-siRNAs is shown in A, and the 5’first nucleotide of ra-siRNAs is shown in B. Transposon, retrotransposon, rRNA and simple repeat represent different derivations of ra-siRNAs.

The length distribution of major classes of ra-siRNAs was shown in Fig. 4A. The 24 nt ra-siRNAs were predominant, corresponding to 52–56% ra-siRNAs from DNA transposons, retrotransposons or simple repeats. By contrast, no obvious length bias was observed among rRNA-derived ra-siRNAs. Moreover, analysis of the 5’ first nucleotide revealed a higher frequency of A than other residues at the 5’end of ra-siRNAs (Fig. 4B). These characteristics are consistent with that found in other plants [34,73].

### PYTs and pha-siRNAs in Pima

**Figure 5:**
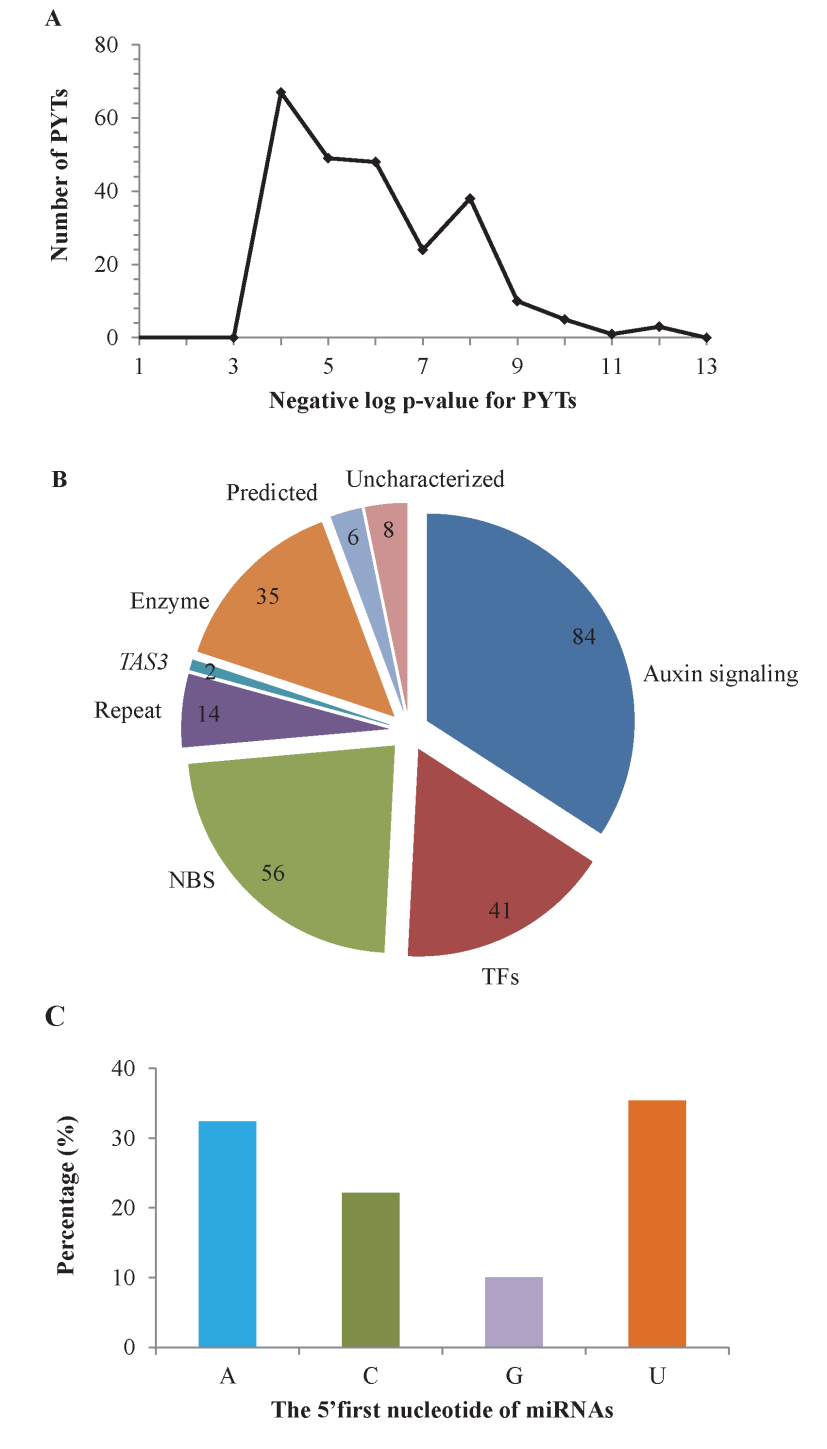
Characteristics of Pima pha-siRNAs. The distribution of p-value for Pima PYTs is shown in A, the classification of PYTs is shown in B, and the distribution of the 5’first nucleotide is shown in C. Auxin: auxin-related transcription factors; Other TF: other transcription factors; NBS: NBS-LRR disease resistance proteins; Repeat: repeat proteins; *TAS3*: *Arabidopsis TAS3* homologs; Enzymes: various enzymes; Predicted: predicted proteins; Uncharacterized: uncharacterized proteins.

Analysis of pha-siRNA was conducted using a combination of UEA Small RNA Workbench [62] and in-house Perl scripts as described in the Method section. As a result, 246 PYTs that produced 627 pha-siRNAs were obtained with significant p-values ranging from 0 to 10^-3^ (Fig. 5A), indicating reliable capture of phasing phenomenon [63].

The PYTs were further annotated using either Blastx against NCBI nr protein database or Blastn against TAIR gene database. Consequently, 236 PYTs were derived from protein coding genes (S5 Table and Fig. 5B), 2 were from non-coding *TAS*3 homologs, and 8 had no hit in the databases. Genes encoding auxin response factors (ARFs) and auxin signaling F-box were the richest source of Pima pha-siRNAs, followed by those encoding NBS-LRR disease resistance proteins and transcription factors. In addition, PYTs were also produced from genes encoding various proteins or enzymes, such as pentatricopeptide repeats, Ca^2+^ ATPase, Helicase, and peroxidase. Strikingly, a DCL1 isoform was able to produce pha-siRNAs (S6 Table), suggesting that the sRNA biogenesis machinery is subject to pha-siRNA regulation [38]. A subset of those PYT genes, which encode proteins, such as auxin signaling F-boxs, pentatricopeptide repeats, NBS-LRR and MYBs, *et al*., has been previously reported in other plants [23,38,74], indicating that these pha-siRNA pathways are likely conserved across plants. Furthermore, analysis of the 5’first nucleotide revealed a higher frequency of A and U and a lower occurrence of C and G at the 5’ end of pha-siRNAs (Fig. 5C). This is consistent with characteristics of DCL products, such as miRNAs and ra-siRNAs.

**Figure 6:**
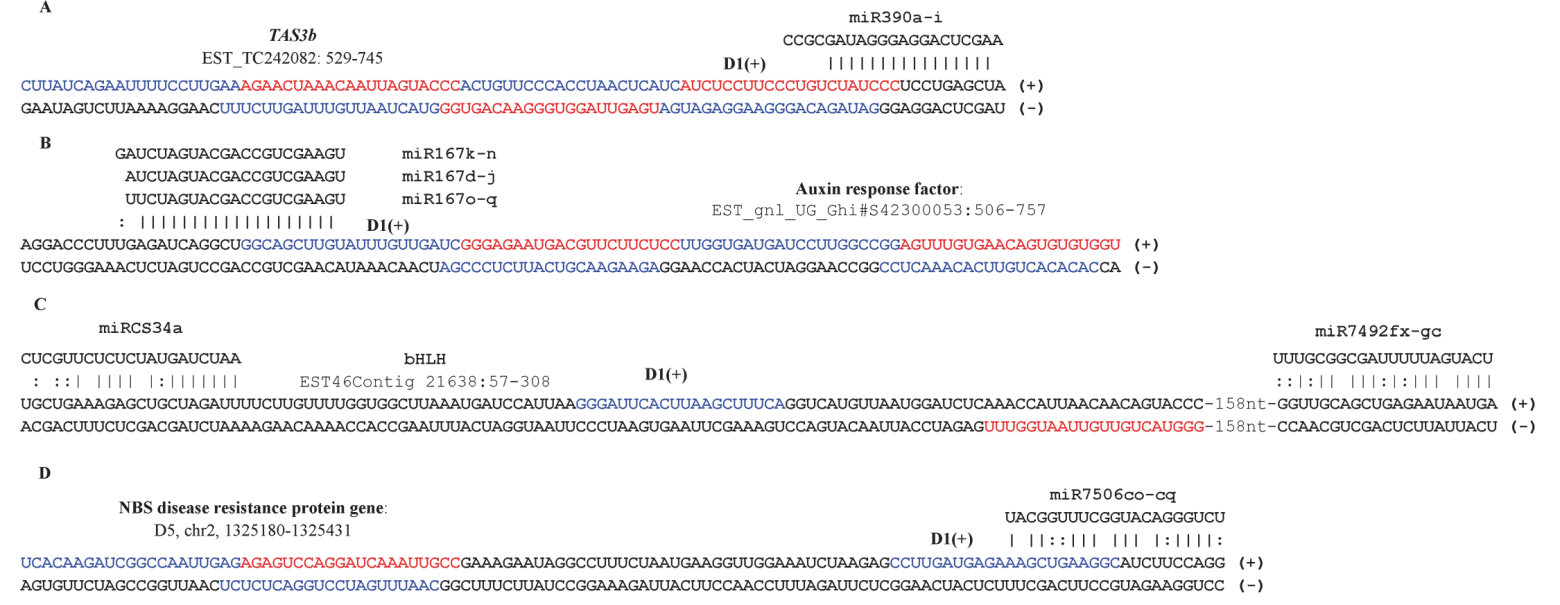
miRNA-mediated pha-siRNA yielding pathways. Four miRNA-mediated pha-siRNAs yielding pathways are shown in A, B, C and D. The red and blue letters represent pha-siRNAs. The vertical line “|” and colon “:” represent Watson-Crick base-pairs and G-U pairs, respectively.

The biogenesis of most known pha-siRNAs is dependent on miRNA cleavage [35,75]. To better understand regulatory pathways of Pima pha-siRNAs, an analysis of phase-initiators was performed, referring to the methodology described previously [64]. As a consequence, 58 miRNA initiators were predicted to target 103 PYTs (S6 Table), including known miRNA-target pairs, such as miR390-*TAS*3 (Fig. 6A) [75,76] and miR167-ARF (Fig. 6B) [77]. However, other miRNA-target pairs, such as miRCS34/miR7492-bHLH (Fig. 6C) and miR7506-NBS-LRR (Fig. 6D), were firstly reported in plants, indicating that they are likely novel pha-siRNA regulatory pathways in plants. Except for the miRCS34/miR7492-bHLH pathway, most PYTs in Pima were triggered by “one-hit” model [35].

### *Cis*-nat siRNAs in Pima

*Cis*-nat siRNAs have been identified in various plants and are shown to regulate plant development, disease resistance and stress response [40,41,78]. However, no *cis*-nat siRNA is reported in Pima to date, although some cotton *trans*-NATs are deposited in plant NAT database, PlantNATsDB (http://bis.zju.edu.cn/pnatdb/) [79]. In this study, 42,737 and 24,178 *cis*-NATs (S7 Table) were identified from cotton A2 and D5 genome, respectively. These *cis*-NATs yielded a total of 136,600 *cis*-nat siRNAs (S8 Table), representing 229,435 reads.

**Figure 7:**
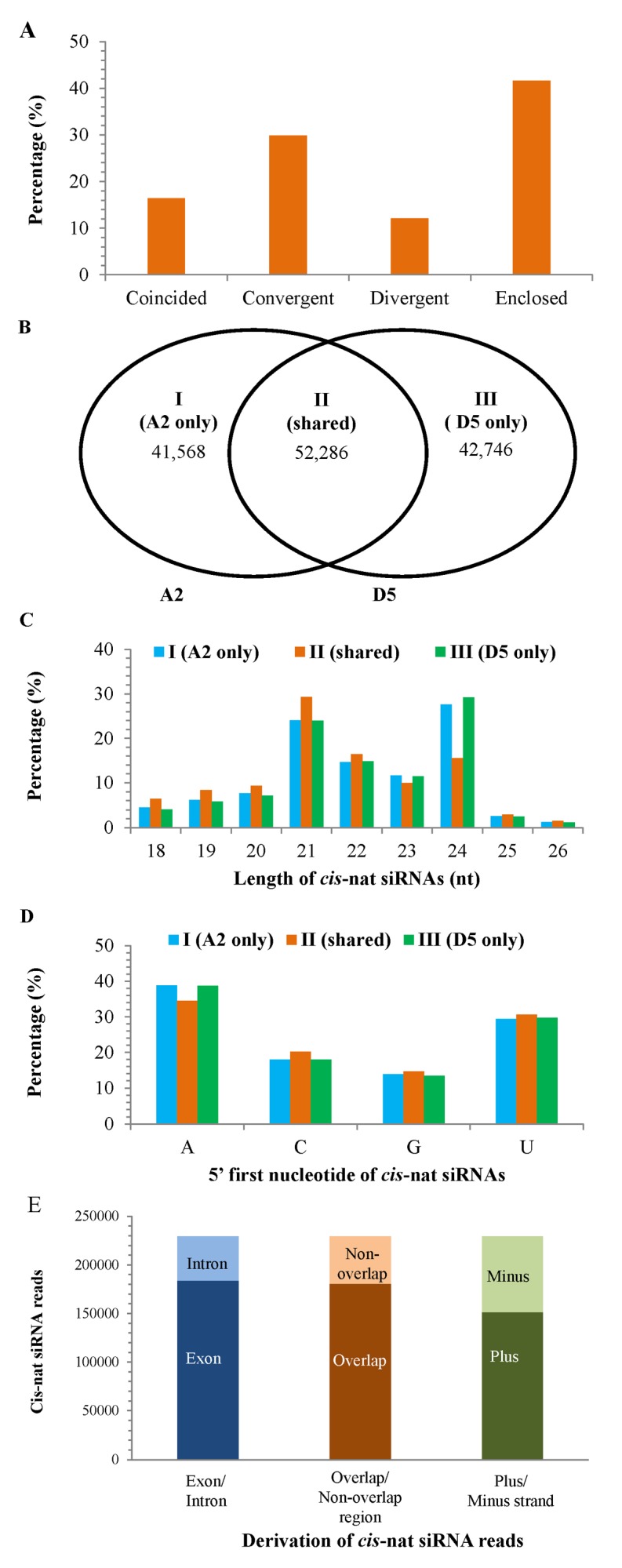
Characteristics of *cis*-NATs and *cis*-nat siRNAs in Pima. The numbers of each type of *cis*-NAT orientations are shown in A. The number of *cis*-nat siRNAs detected from the A2 and D5 genome is shown in B. The length distribution of *cis*-nat siRNAs is shown in C. The 5’first nucleotide of *cis*-nat siRNAs is shown in D. The enrichment analysis of *cis*-nat siRNAs is shown in E; exon/intron, overlap/non-overlap region, and plus/minus stand represent different derivations of *cis*-nat siRNAs.

The *cis*-NATs were further classified into 4 types in accordance to overlapping orientations: convergent (3’end overlap), divergent (5’end overlap), enclosed (one transcript encompassed the other transcript), and coincided (two transcripts of *cis*-NATs completely overlapped). Most *cis*-NATs hybridized in enclosed and convergent orientations, corresponding to 42% and 30% of the total *cis*-NATs, respectively, while only 10–16% overlapped in the divergent or coincided way (Fig. 7A). Compared to earlier studies [41,78], *cis*-NATs in Pima can form dsRNAs in the coincided orientation, a new overlapping means found in plants. In addition, nearly half *cis*-NATs in Pima overlapped in the enclosed way, differing from that the convergent orientation is the major overlapping means found previously [41,78], which reflects the overlapping means of *cis*-NATs might vary in different plant species or tissue types.

Of the 136,600 *cis*-nat siRNAs, 38% (52,286) were expressed in both A2 and D5 genomes (Fig. 7B), while 41,568 (31%) and 42,746 (31%) were uniquely detected in A2 and D5 genome (Fig. 7B), respectively. Interestingly, *cis*-nat siRNAs uniquely detected in the A2 and D5 genome shared a similar length distribution, with the 24 nt *cis*-nat siRNAs (~29%) being the most abundant (Fig. 7C). However, those shared between the two genomes displayed a different length characteristic with the 21 nt *cis*-nat siRNAs (29%) being predominant (Fig. 7C). Moreover, the *cis*-nat siRNAs exhibited a 5’first nucleotide bias for A and U (Fig. 7D). These are consistent with previous studies in plant *cis*-nat siRNAs [65,73,78].

Furthermore, analysis of enrichment of *cis*-nat siRNAs was conducted by mapping sRNAs to the *cis*-NAT genomic loci, using Bowtie Mapping Utility [80]. The results revealed that most *cis*-nat siRNA reads were produced from exons (80%) or overlapping regions (79%) (Fig. 7E). In addition, 66% *cis*-nat siRNA reads were produced from plus strands (Fig. 7E), approximately two folds of that from minus strands, reflecting that *cis*-nat siRNAs exhibited strand-biased expression [41,73,78].

### *Trans*-nat siRNAs in Pima

**Figure 8:**
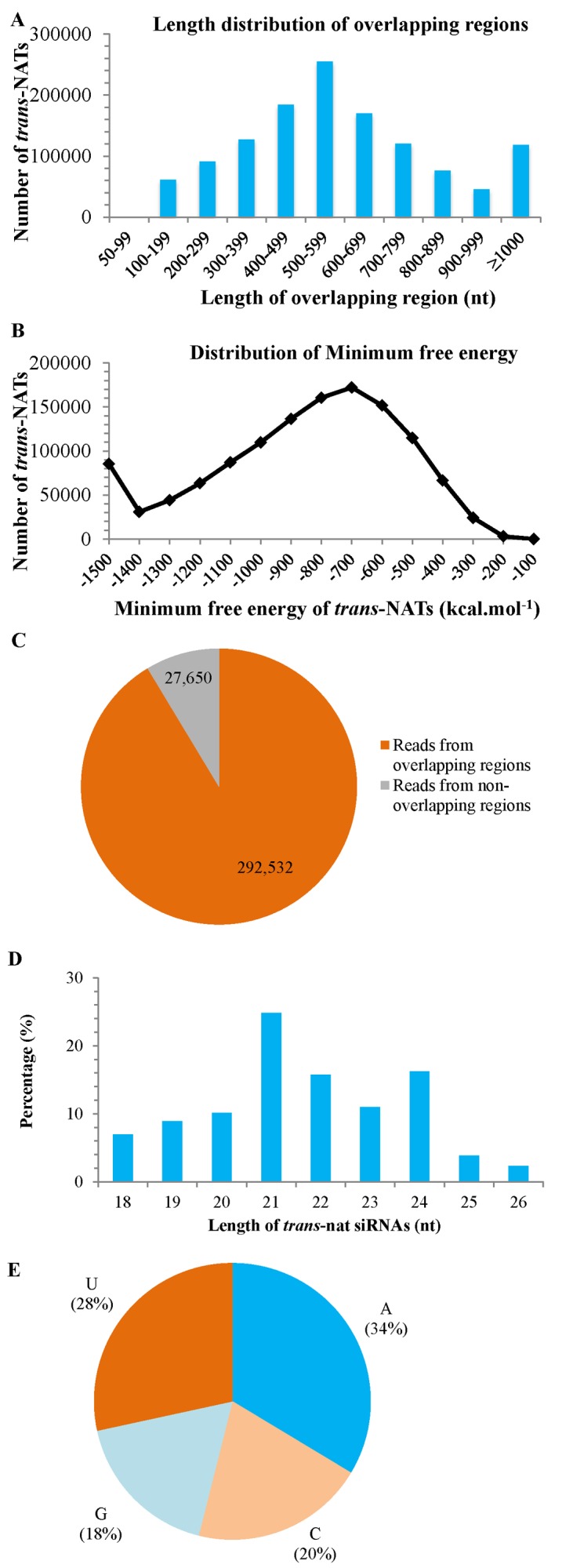
Characteristics of *trans*-NATs and *trans*-nat siRNAs. The length of overlapping regions of *trans*-NATs is shown in A. The distribution of minimal free energy of *trans*-NATs is shown in B. The enrichment analysis of *trans*-nat siRNAs along *trans*-NATs is shown in C. The distribution of length of *trans*-nat siRNAs is shown D. The 5’first nucleotide of *trans*-nat siRNAs is shown in E.

In this study, 1,250,581 *trans*-NATs were identified in Pima, of which 895,809 (72%) were detected to yield 79,994 *trans*-nat siRNAs (S9 Table). These *trans*-NATs overlapped in a wide length range from 50 to more than 1000 nt (Fig. 8A), while most (68%) had an overlapping region between 300 and 800 nt. The *trans*-NATs can form dsRNAs with a minimum free energy ranging from -1500 to -100 kcal.mol^-1^ (Fig. 8B), of which 85% ranged from -1200 to -400 kcal.mol^-1^, indicating that these *trans*-NATs can form stable dsRNAs.

The 79,994 *trans*-nat siRNAs represented a total of 320,182 reads (Fig. 8C), and 91% reads were derived from overlapping regions. *Trans*-nat siRNAs ranged from 18 to 26 nt in length, with the 21 nt siRNAs being the most abundant (Fig. 8D). Most *trans*-nat siRNAs (62%) had an A or U as the 5’first nucleotide (Fig. 8E), and relatively fewer G and C appeared at the most 5’end.

To date, *trans*-NATs have been identified in a broad range of plants [79], such as Upland cotton and *G*. *raimondii*, while no *trans*-nat siRNA has been previously reported in Pima. In this study, we firstly identified a large number of *trans*-NATs in Pima, producing a significant number of *trans*-nat siRNAs. Consistent with the major length range of DCL products [10,81], most *trans*-nat siRNAs were 21 to 24 nt in length and had the characteristic of the 5’first nucleotide similar to other sRNAs in Pima, indicating that they are likely products of DCL cleavage. Like *cis*-nat siRNAs and in agreement with previous study [73], most *trans*-nat siRNA reads were produced from the overlapping regions, suggesting that a similar mechanism might govern the synthesis of NAT-derived siRNAs.

## Conclusion

Through analysis of deep sequencing sRNA data of Pima, 5 major classes of sRNAs including miRNAs, ra-siRNAs, pha-siRNAs, *cis*-nat and *trans*-nat siRNAs along with their precursors were bioinformatically identified. This is the first comprehensive analysis of sRNAs in Pima and creates an important molecular resource for future studies on mechanism of regulating plant growth and development.

## Supporting Information

S1 Table Summary for the two small RNA libraries of Pima (*G*. *barbadense*). (XLSX)

S2 Table Pima miRNAs and their pre-miRNAs. (XLSX)

S3 Table Protein-coding gene derived pre-miRNAs. (XLSX)

S4 Table ra-siRNAs with abundance ≥10 reads. (XLSX)

S5 Table Pima phased siRNAs and phased siRNA-yielding transcripts. (XLSX)

S6 Table Predicted miRNA initiators for pha-siRNAs. (XLSX)

S7 Table *cis*-NATs identified in the cotton A2 and D5 genome. (XLSX)

S8 Table *cis*-nat siRNAs in Pima. (XLSX)

S9 Table *tran*s-nat siRNAs in Pima. (XLSX)
